# Kallikrein 6 protease advances colon tumorigenesis *via* induction of the high mobility group A2 protein

**DOI:** 10.18632/oncotarget.27153

**Published:** 2019-10-22

**Authors:** Hwudaurw Chen, Earlphia Sells, Ritu Pandey, Edward R. Abril, Chiu-Hsieh Hsu, Robert S. Krouse, Raymond B. Nagle, Georgios Pampalakis, Georgia Sotiropoulou, Natalia A. Ignatenko

**Affiliations:** ^1^ University of Arizona Cancer Center, Tucson, AZ, USA; ^2^ Biochemistry and Molecular and Cellular Biology Graduate Program, Department of Molecular and Cellular Biology, College of Science, University of Arizona, Tucson, AZ, USA; ^3^ Department of Cellular and Molecular Medicine, University of Arizona, Tucson, AZ, USA; ^4^ Mel and Enid Zuckerman College of Public Health, University of Arizona, Tucson, AZ, USA; ^5^ University of Arizona College of Medicine, Tucson, AZ, USA; ^6^ Southern Arizona Veterans Affairs Health Care System, Tucson, AZ, USA; ^7^ Department of Pathology, College of Medicine, University of Arizona, Tucson, AZ, USA; ^8^ Department of Pharmacy, University of Patras, Patras, Greece

**Keywords:** colorectal cancer, kallikrein-related peptidase 6 or KLK6, SMAD2/3, epithelial-mesenchymal transition, HMGA2

## Abstract

Kallikrein-related peptidase 6 (KLK6) overexpression is commonly observed in primary tumors of colorectal cancer (CRC) patients and has been associated with tumor aggressiveness, metastasis, and poor prognosis. We previously established a unique contribution of KLK6 in colon cancer metastasis via a specific network of microRNAs and mRNAs. Here we evaluated the cellular functions of KLK6 protease in Caco-2 colon adenocarcinoma cell line after introduction of the enzymatically active or inactive form of the enzyme. We found that proteolytically active KLK6 increased Caco-2 cells invasiveness *in vitro* and decreased the animal survival in the orthotopic colon cancer model. The active KLK6 induced phosphorylation of SMAD 2/3 proteins leading to the altered expression of the epithelial-mesenchymal transition (EMT) markers. KLK6 overexpression also induced the RNA-binding protein LIN28B and high-mobility group AT-hook 2 (HMGA2) transcription factor, two essential regulators of cell invasion and metastasis. In the CRC patients, KLK6 protein levels were elevated in the non-cancerous distant and adjacent tissues, compared to their paired tumor tissues (*p* < 0.0001 and *p* = 0.0157, respectively). Patients with mutant K-RAS tumors had significantly higher level of KLK6 protein in the luminal surface of non-cancerous distant tissue, compared to the corresponding tissues of the patients with K-RAS wild type tumors (*p* ≤ 0.05). Furthermore, KLK6 and HMGA2 immunohistochemistry (IHC) scores in patients’ tumors and paired adjacent tissues positively correlated (Spearman correlation *P* < 0.01 and *p* = 0.03, respectively). These findings demonstrate the critical function of the KLK6 enzyme in colon cancer progression and its contribution to the signaling network in colon cancer.

## INTRODUCTION

Human KLK6 (initially named protease M/zyme/neurosin), is a member of the kallikrein-related peptidase family of proteins, originally identified and cloned based on its aberrant expression in human breast and ovarian cancer [1]. KLK6 is a secreted serine protease synthesized as a proenzyme. The zymogen (proKLK6) is activated by limited proteolysis either autolytically [2–4] or, possibly, by another unidentified protease [5].

Various evidence implicate KLK6 in human cancers [1, 6–17]. As a proteolytic enzyme, KLK6 can contribute to the invasive phenotype of cancer cells *via* degradation of extracellular matrix proteins, such as collagen, fibronectin, laminin, fibrinogen and activation of matrix metalloproteinases [6, 10]. KLK6 has been reported to facilitate cell migration and invasion via its effects on the epithelial-mesenchymal transition (EMT). EMT is a fundamental process of cellular phenotypic transitions during embryonic development, as well as in wound healing and neoplastic transformation [18]. When KLK6 was overexpressed in mouse keratinocytes and HEK293 cells, an upregulation of the EMT marker vimentin and loss of E-cadherin was observed [10]. On the contrary, re-expression of KLK6 in non-expressing breast cancer cell lines resulted in suppression of their malignant phenotypes through inhibition of vimentin, upregulation of calreticulin and epithelial markers cytokeratin 8 and 19 [11]. Similar inhibitory function of KLK6 on the EMT markers was reported in head and neck squamous cell carcinoma [15].

In colon cancer, correlation was established between elevated KLK6 expression and secretion and aggressive tumor behavior and poor patient outcome [9, 12, 13]. The KLK6 transcript was identified as one of 12 biomarkers for poor prognosis in patients with stage II CRC [8]. KLK6 overexpression was reported in precancerous colorectal and duodenal adenomas and early stage adenocarcinomas with an upregulated Wnt/β-catenin pathway [19, 20]. We previously reported that introduction of the mutated K-RAS oncogenic driver gene into Caco-2 colon cell line, which express wild type K-RAS, induced KLK6 expression [7, 14]. Knocking down endogenously overexpressed KLK6 in highly invasive HCT116 cells, which carries K-RAS mutation (*K-RAS^G12D^*), was sufficient to decrease the invasive and metastatic properties of this cell line [17]. These findings provide reason for further investigation of the role of the KLK6 enzyme in colon cancer progression. In the present study we investigated the consequences of KLK6 overexpression and its enzymatic activity in colon cancer cells. We found that KLK6 overexpression in colon cancer cells, regardless of its enzymatic activity, induces the expression of transcription associated protein HMGA2, which has been identified as a driver of the CRC progression and metastasis [21, 22]. We also observed induction of the SMAD phosphorylation and expression of EMT markers associated with expression of the active KLK6 enzyme.

## RESULTS

### Overexpression of enzymatically active KLK6 increases tumorigenicity of Caco-2 colon cancer cells

**Figure 1:**
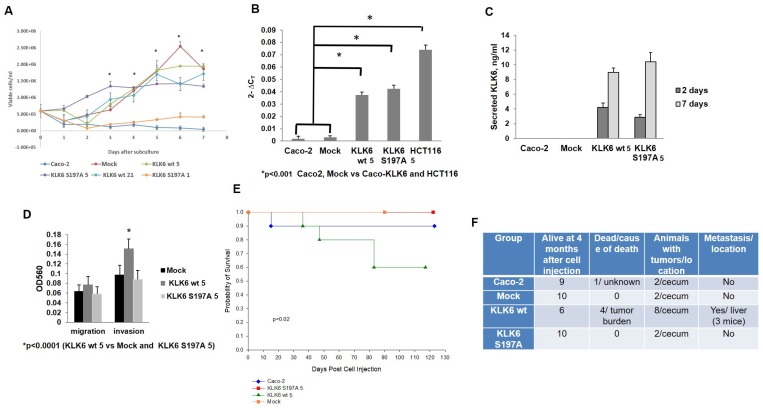
Characterization of Caco-KLK6 cell model. (**A**) Growth curve of Caco-2 and Caco-KLK6 stable isogenic clones. ^*^ *p <* 0.02 (Caco-2 & KLK6 S197A 1 vs Mock, KLK6 wt 5, KLK6 wt 21 and KLK6 S197A 5, by ANOVA. Figure is representative of two independent experiments run with triplicate samples with error bars indicating SD. (**B**) KLK6 transcript levels by qPCR in Caco-2 parental cells and Caco-2 stable isogenic clones. The level of KLK6 in HCT116 cells is shown as a reference. Analysis was done 48 hours after subculture. ^*^ *p <* 0.001 by ANOVA. (**C**) Levels of secreted KLK6 in conditioned media of Caco-2 parental cells, Mock cells and Caco-2 isogenic clones at 2 and 7 days after subculture by KLK6 ELISA. (**D**) Invasion through Matrigel depends on the KLK6 enzymatic activity. ^*^ *p <* 0.0001 by ANOVA, KLK6 wt 5 *vs* Mock and KLK6 S196A 5. (**E**) Kaplan–Meier survival curve of SCID mice injected with Caco-2 cells and Caco–KLK6 isogenic clones (*n* = 10 mice per group; *P =* 0.02 by long-rank test). (**F**) Analysis of tumor incidence and location in Caco-2 SCID orthotopic colon cancer model. (*N =* 10 animals per group).

In order to study the contribution of the KLK6 enzyme in cell invasion, we chose the non-invasive Caco-2 cell line, which does not express and secrete KLK6 protein (Supplementary Figure 1A–1C). In contrast, the HCT116 cell line (*K-RAS^G12D^*) overexpresses KLK6 and is highly invasive *in vivo.* (Supplementary Figure 1 and [17]). We transfected Caco-2 cells with the enzymatically active wild-type KLK6 (KLK6 wt plasmid) and inactive KLK6 (KLK6 S197A or mutant plasmid). The KLK6 S197A plasmid, which carries active site serine to alanine mutation at residue 197, has been previously constructed and characterized [3, 11]. After 6 weeks of passaging in selection media, four clones of Caco-2 transfected with an empty vector (Mock cells), seven clones of KLK6 wt expressing cells, and four clones of KLK6S197A expressing cells were developed. Growth rates of these isogenic clones were initially measured to determine whether exogenous overexpression of KLK6 altered cell growth. No significant difference was observed in growth rates of Mock, KLK6 wt clone 5 (KLK6wt 5) and KLK6 S197A clone 5 (KLK6 S197A 5) (4 days doubling time). These clones grew faster than Caco-2 parental cells and KLK6 S197A 1 clone (6 days doubling time) (Figure 1A). There were no apparent changes in the cellular morphology of the Caco-2 stable clones and parental cells (data not shown).

### mRNA levels, protein expression and secretion levels in Caco-2 stable clones

KLK6 expression in the Caco-2 KLK6 isogenic clones (Caco-KLK6 model) was validated at the RNA and protein level and compared to Caco-2 parental cells. KLK6 wt 5 and KLK6 S197A 5 clones expressed high levels of KLK6 transcript, while KLK6 transcript was not detectable in the Mock clone and parental Caco-2 cells at 48 hours (Figure 1B). Similarly, protein secretion was higher in KLK6 wt 5 and KLK6 S197A 5 clones at all time points compared to the Mock clone and Caco-2 parental cells (Figure 1C, levels of secreted KLK6 on days 2 and 7 are shown).

### Migration and invasion of Caco stable clones *in vitro*

Migration rates were similar for Caco-2 cells overexpressing KLK6 either wt or inactive KLK6 protein, but KLK6 wt 5 cells invaded through the Matrigel at the significantly higher rates compared to Mock and KLK6 S197A 5 clones (*p* < 0.0001) (Figure 1D).

### Effect of KLK6 over-expression in an orthotopic colon cancer mouse model

We further tested the consequences of expressing enzymatically active and inactive *KLK6* in Caco-2 isogenic clones *in vivo* in an SCID orthotopic colon cancer model (*N =* 10 mice per group). Mice were monitored weekly, and sacrificed when they became morbid. Caco-2 cells are known for their very low tumorigenic potential *in vivo* as shown in Supplementary Figure 1D and [7], therefore tumor growth was monitored for 4 months. During the course of the experiment, 4 out of 10 animals, which were injected with KLK6 wt 5 clone became morbid and were sacrificed earlier due to complications from the primary tumor, in contrast to animals injected with Caco-2 parental, Mock or KLK6 S197A 5 cells. Kaplan–Meier survival analysis confirmed a significant decrease in survival of animals injected with KLK6 wt 5 clone (Figure 1E, *P* = 0.02). Postmortem analysis showed that 80% of mice injected with KLK6 wt 5 clone developed tumors and 37.5% of tumor-bearing mice produced liver metastasis (Figure 1F and Supplementary Figure 2). Thus, Caco-2 colon adenocarcinoma cell line acquired the invasive and metastatic properties upon overexpression of active KLK6 protease. Animals, injected with KLK6 wt 5 cells, had a significantly shorter survival time (median survival time for KLK6 wt-injected mice was 82 days in contrast to 120 days survival time for other groups).

### Overexpression of active KLK6 enzyme in Caco-2 cell line leads to phosphorylation of SMAD proteins

**Figure 2:**
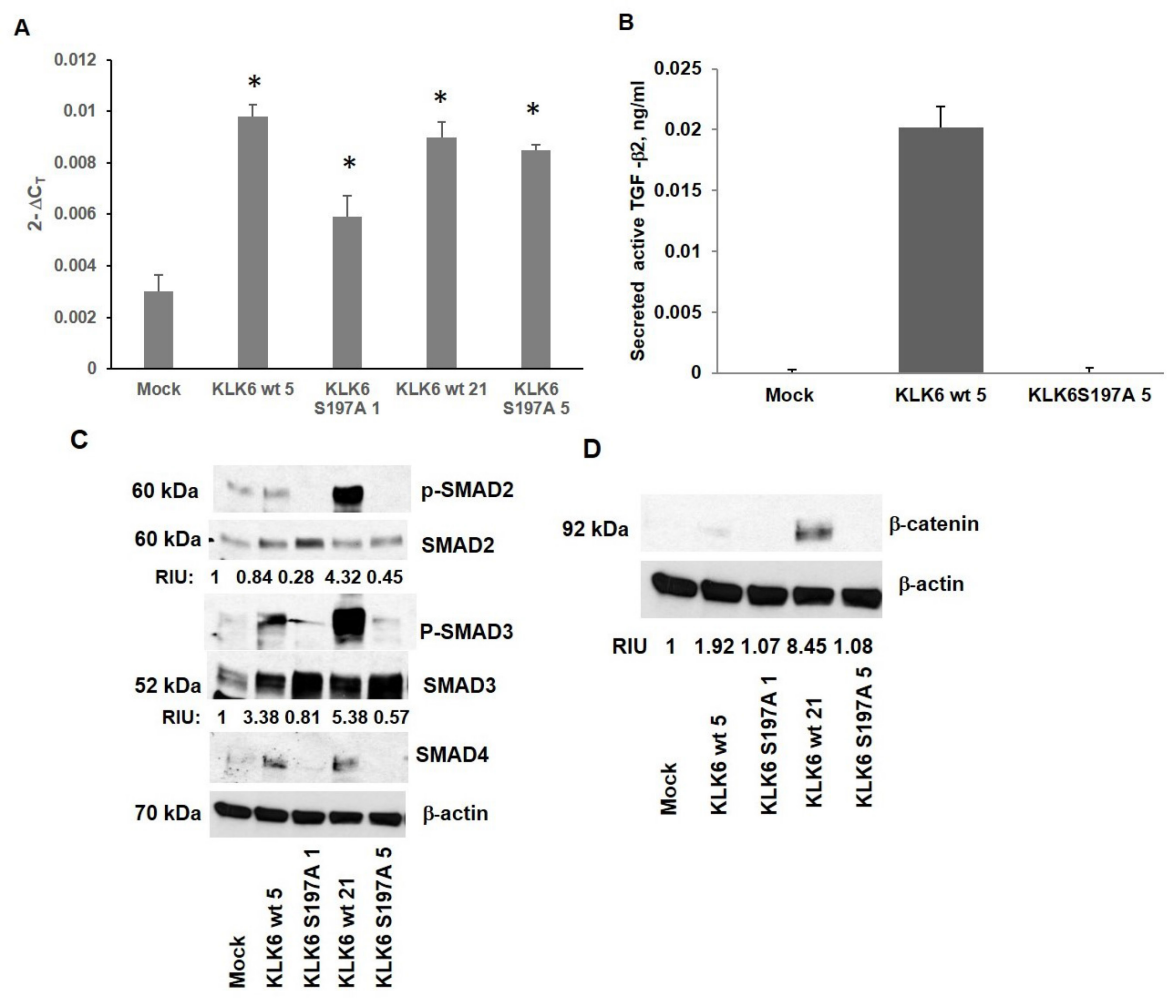
Analysis of SMAD signaling and β-catenin expression in Caco-KLK6 isogenic clones (**A**) The level of TGF-β2 transcript in Caco-KLK6 isogenic clones by qPCR; ^*^ *p <* 0.02 (Mock vs Caco-KLK6 clones wt clones 5 and 21, KLK6 S197A clones 1 and 5, by ANOVA). (**B**) The level of active TGF-β2 ligand in the conditioned media of Caco-2 isogenic clones expressing the active KLK6 protease (KLK6 wt 5) and mutant KLK6 (KLK6 S197A 5). (**C**) Analysis of intracellular levels of SMAD proteins in Caco-KLK6 stable isogenic clones by Western blotting. (**D**) Analysis of the β-catenin expression in Caco-KLK6 isogenic clones. RIU**:** relative intensity units, quantification was done using Image J. Bands of phosphorylated SMAD2/3 proteins were normalized to the levels of total SMAD2/3 protein; bands of β-catenin were normalized to β-actin. The protein sizes are indicated in kDa. Figure is a representative of two independent experiments.

Our previous studies with knockdown of KLK6 in HCT116 cells determined that KLK6 controls cell invasion and metastasis via a microRNA-mRNA network, which includes the Transforming Growth Factor β2 (TGF-β2) isoform pathway [17]. Here we evaluated TGF-β2 expression and secretion in Caco-KLK6 clones. TGF-β2 transcript level was measured in two KLK6 wt clones and two KLK6 mutant clones and was found to be significantly elevated compared to its level in the Mock transfected clone (2-3-fold increase, Figure 2A, *p* < 0.02). The secreted active TGF-β2 protein was measured in the conditioned media of Mock transfected, KLK6 wt 5 and KLK6 S197A 5 clones (clones used for i*n vivo* experiment above). Active TGF-β2 protein was detected only in the media from KLK6 wt 5 clone, but not from Mock or KLK6 S197A 5 clones (Figure 2B). This finding suggests that KLK6 overexpression may contribute to TGF-β2 production in CRC, and KLK6 protease activates the latent form of TGF-β protein.

We further assessed SMAD signaling in Caco-2 isogenic clones (Figure 2C). The phosphorylated SMAD2 protein (Ser465/467) was induced by more than 4-fold in both KLK6 wt clones when compared to the mutant KLK6 expressing clones. The level of phosphorylated SMAD2 varied between KLK6 wt clones when compared to the Mock clone (phosphorylated SMAD2 level in KLK6 wt 5 clone was not altered while KLK6wt 21 clone produced 4 times more phosphorylated SMAD2 protein than Mock clone). The phosphorylated SMAD3 protein (Ser423/425) was elevated by 3-5-fold in both clones expressing the functional KLK6 enzyme (KLK6 wt 5 and KLK6 wt 21 clones), compared to Caco-2 Mock clone. Elevated expression of the SMAD4 protein was also observed in Caco-2 clones with active KLK6. In Caco-2 cell line, one allele of the SMAD4 gene is lost and the other has point mutation (*SMAD4 ^-/D315H^*) located in MH2 domain, responsible for protein homo- and heterodimerization, causing SMAD4 to be non-responsive to TGF-β stimulation [23]. TGF-β ligands are capable of activating both canonical, receptor-associated SMAD signaling and non-SMAD signaling via Rho, RAC, MEK1/2, ERK1/2, AKT-mTOR and other pathways [24], which may cause an increase in SMAD4 protein level in the enzymatically active KLK6 Caco-2 clones. To address this possibility, we measured expression of the phosphorylated extracellular signal-regulated kinases ERK1 and ERK2 in Caco-2 KLK6 clones and found a 3-fold increase in ERK1/2 level in KLK6 wt 5 cells, compared to Mock and KLK6 S197A5 clones (Supplementary Figure 3).

Additionally, because we observed activation on SMAD signaling upon expression of KLK6 protease (Figure 2A, 2B), we evaluated the status of the EMT markers and EMT-inducing transcription factors in Caco-KLK6 model. We found elevated level of β-catenin in KLK6 wt clones (2-fold induction in KLK6 wt clones 5 and 8.5- fold induction in KLK6 wt 21 clone) with no significant expression of β-catenin in KLK6 mutant clones (Figure 2D).

Expression of N-cadherin, vimentin, and Slug1 proteins was not detected in these cells. The levels of E-cadherin, Snail and ZEB1 proteins were not significantly altered in Caco-KLK6 cell model (data not shown).

### Transient re-expression of KLK6 in HCT116 cell model with KLK6 knockdown restores the invasive phenotype and the EMT features of HCT116 cell line

In HCT116 colon cancer cells with KLK6 knockdown (shKLK6 cells) TGF-β2 was identified as one of the top downregulated genes [17]. Here we confirmed a 3-4-fold suppression of TGF-β2 transcript level in shKLK6 clones 2, 3, and 4, compared to the Control clone 1 (Supplementary Figure 4A, *p* ≤ 0.02 in shKLK6 isogenic clones by ANOVA). Next, we evaluated the level of SMAD proteins in shKLK6 clones. We found that shKLK6 clones had lower level of the phosphorylated SMAD2 protein (2-4 fold decrease in the tested shKLK6 clones) compared to HCT116 Control clone 1. Levels of total SMAD2, SMAD3 and SMAD4 were unchanged upon knockdown of KLK6 expression in HCT116 cells (Supplementary Figure 4B).

**Figure 3:**
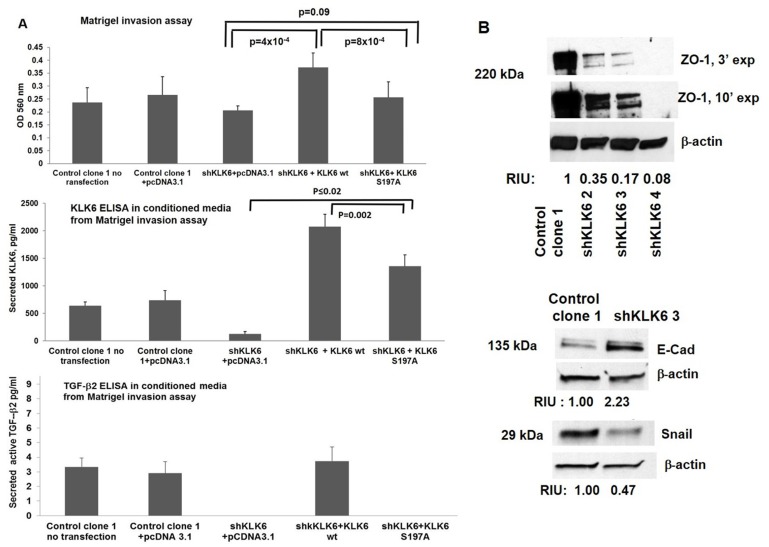
KLK6 enzyme contributes to cell invasion in HCT116-shKLK6 model through induction of the EMT. (**A**). KLK6 enzymatic activity is required for invasive phenotype of HCT116 cells. Upper panel: Cell invasion through Matrigel following transient transfection of KLK6 wt or KLK6 S197A plasmids into HCT116 control clone 1 and shKLK6 clone 3. Cells were seeded in Matrigel invasion chambers 24 h after transfection and were analyzed 48 h later. HCT116 Control clone 1 untransfected and co-transfected with pcDNA3.1 vector served as controls. ^*^ *p =* 0.0004 (shKLK6 +pcDNA3.1 vs shKLK6+wt KLK6), ^**^ *p =* 0.0008 (shKLK6-3/KLK6 wt vs shKLK6/KLK6) S197A, by *t*-test. This figure is representative of two independent experiments performed in sextuplets with error bar indicating SD. Middle panel: Secreted KLK6 protein levels in conditioned media by ELISA from Matrigel invasion assay (upper panel), ^***^ *p =* 0.0002 and ^**^ *p =* 0.0008 (shKLK6+wt KLK6 vs shKLK6+KLK6 KLK6 S197A), by paired *t*-test, respectively. Lower panel. Analysis of secreted active TGF-β2 protein in the conditioned media from Matrigel invasion assay. Figures are representative of two independent experiments performed with triplicates. Error bars indicate SD. (**B**) Levels of ZO1, E cadherin and Snail in HCT-shKLK6 cell model. RIU: relative intensity units, quantification was done using Image J. Bands of the protein of interest were normalized to β-actin. The proteins sizes are indicated in kilodalton (kDa). Images are representative of three independent experiments.

We performed a KLK6 rescue experiment in HCT116 cell line with KLK6 knockdown by transiently transfecting one of the shKLK6 isogenic clones (shKLK6 clone 3) with KLK6 wt and KLK6 S197A plasmids. Cell invasion, levels of secreted KLK6 and active TGF-β2 proteins in the conditioned media were assessed at 48 hours after the transfected cells were seeded in the invasion chambers. We observed a significant increase in invasion of shKLK6 3 cells transfected with KLK6 wt plasmid compared to the cells transfected with pcDNA3.1(+) empty plasmid (*p =* 0.0004) and KLK6 S197A plasmid (*p =* 0.0008; Figure 3A Upper panel). Cell migration rates were compatible in shKLK6 3 cells, which expressed enzymatically active KLK6 and inactive KLK6 (data not shown). Levels of secreted KLK6 were elevated in the conditional media from both shKLK6 wt and shKLK6 S197A cells compared to shKLK6 cells expressing pcDNA3.1(+) empty plasmid (*p* ≤ 0.02; Figure 3A Middle panel). Secreted active TGF-β2 protein was detected only in the conditioned media from shKLK6 3 cells transfected with KLK6 wt plasmid (Figure 3A Lower panel).

We further assessed whether transient re-expression of enzymatically active or mutant KLK6 altered SMAD proteins expression and phosphorylation. Three shKLK6 isogenic clones were transiently transfected with KLK6 wt or KLK6 S197A plasmids and the levels of total and phosphorylated SMAD proteins were measured by Western blotting. We found that SMAD2 protein levels (both total and phosphorylated proteins) were elevated only in shKLK6 clones expressing active KLK6 protease (Supplementary Figure 4C). Phosphorylated SMAD3 protein was detected in shKLK6 clones 2 and 3 when KLK6 wt is expressed (Supplementary Figure 4D). In shKLK6 clone 4 both active and inactive KLK6 induced SMAD3 expression and phosphorylation. Total SMAD3 was not altered in KLK6 wild type and mutant cultures. The observed variability among shKLK6 clones may be a result of variation in transient transfection.

Similarly, we evaluated the status of the EMT-inducing proteins in HCT116-shKLK6 model. We observed a 3-4 fold reduction in the ZO1 protein level in shKLK6 clones (Figure 3B, upper panel). A 2-fold induction in the level of the epithelial marker E-cadherin and 2-fold suppression of the mesenchymal marker Snail were seen in shKLK6 clone 3 compared to Control Clone 1 (Figure 3B, lower panel). No significant changes in β-catenin levels were seen among KLK6 knockdown clones (data not shown). N-cadherin, vimentin and Slug1 were undetectable in HCT116-shKLK6 model and the ZEB1 level was not altered in the tested isogenic cell lines (data not shown). These results corroborate with our findings of KLK6 effects in Caco-KLK6 cell model.

### LIN28B and HMGA2 expression parallels with KLK6 expression in colon cancer cells

**Figure 4:**
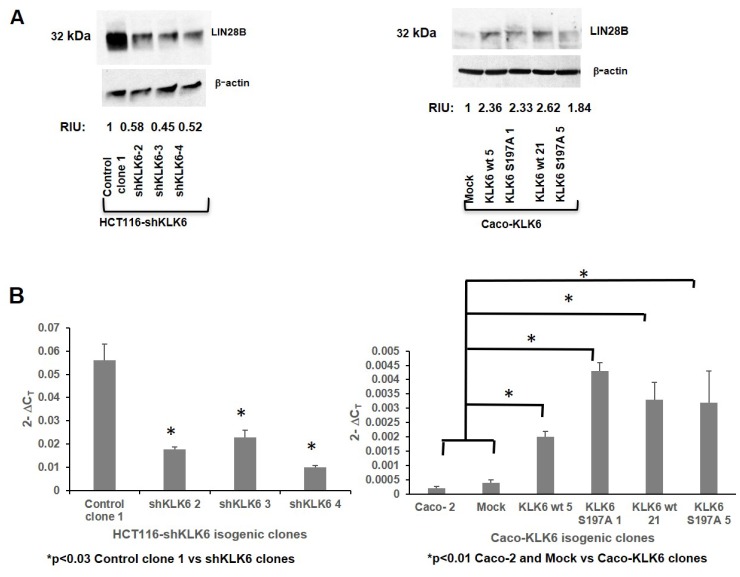
Expression of KLK6 correlates with expression of LIN28B and HMGA2 proteins in colon cancer cells. (**A**) Expression of LIN28B in HCT116-shKLK6 model and Caco-KLK6 cell model by Western blotting. β-actin was used as a loading control. RIU**:** relative intensity units, quantification was done using ImageJ. Bands of the protein of interest were normalized to β-actin. The protein size is indicated in kDa. Figure is representative of two independent experiments. (**B**) Expression of HMGA2 by qPCR in HCT116-shKLK6 and Caco-KLK6 isogenic clones. *P* values were calculated by ANOVA.

We further explored the link between KLK6 protease and regulators of cell invasion and metastasis, such as the LIN28 ribosomal binding protein paralogs, LIN28A and LIN28B, and chromatin-binding protein HMGA2 [25, 26]. The levels of LIN28A protein were unchanged in Caco-2-KLK6 and HCT116-shKLK6 colon cell models (data not shown). However, LIN28B protein level (isoform 1) decreased by approximately 2-fold in all tested shKLK6 clones (Figure 4A, left panel). LIN28B protein level was more than 2-fold higher in Caco-2 clones, which overexpressed KLK6, compared to the Mock clone (Figure 4A, right panel). The upregulation of LIN28B protein in Caco-2 cells was independent of the proteolytic activity of KLK6.

RT-PCR was used to determine the HMGA2 expression levels in HCT116-shKLK6 and Caco-KLK6 clones. In shKLK6 clones HMGA2 transcript level was 2-5-times lower compared to HCT116 Control clone 1 (Figure 4B, left panel, *p* < 0.03). HMGA2 transcript level was significantly higher in all Caco-KLK6 isogenic clones compared to Mock clone (*p* < 0.01, 5- and 8-fold increase in KLK6 wt 5 and KLK6 wt 21, respectively, and 11- and 8-fold increase in KLK6 S197A 1 and KLK6S197A 5, respectively).

### KLK6 expression correlated with HMGA2 expression in the surgical material of CRC patients

To elucidate the relevance of our *in vitro* findings of KLK6 function in colon tumorigenesis we assessed the status of the KLK6 protein by IHC in FFPE tissue sections of colon cancer patients (*N =* 10, described in detail in “Material and Methods” section). The set included a primary tumor and paired tissues at 1 cm and 10 cm away from the tumor. The KLK6 staining pattern was scored by a blinded pathologist and analyzed for statistical significance. KLK6 staining in the crypt area of the tumor samples was significantly higher than in the morphologically normal tissue at 10 cm away from the tumor (Table 1A). The luminal surface area at 1 cm and 10 cm away from the tumor site was stained positively for KLK6 (*p =* 0.016 at 1 cm and *p* < 0.0001 at 10 cm), suggesting the increased KLK6 secretion by tumor cells and/or the presence of KLK6 in the morphologically normal tissue surrounding the tumor as well as in the distant areas. When this analysis was stratified according to the status of the *K-RAS* oncogene in the primary tumor, a significant increase in the KLK6 staining score was observed in the luminal surface and crypts of the non-cancerous tissues 10 cm away from the primary tumors positive for K-RAS mutation (Table 1B, *p =* 0.045 and *p =* 0.0515, respectively). *K-RAS^G12A^* mutation was prevalent in this set of samples, with a frequency of 21.875%. Supplementary Figure 5 illustrates the KLK6 IHC pattern in *K-RAS* wild type and *K-RAS^G12A^* clinical cases.

**Table 1A:** Analysis of KLK6 IHC in clinical material depending on the cellular localization and distance from tumor site (Fisher exact test)

Analysis of KLK6 staining based by Fisher’s exact test (*N* = 10)			
Location	Distance from tumor site	%Negative	%Positive
Crypts	10 cm	60	40
	1 cm	40	60
	tumor^***^	0	100
Luminal surface	10 cm^**^	0	100
	1 cm^*^	10	90
	tumor	80	20

^*^ *p* = 0.0157; 1 cm vs tumor; ^**^ *p* < 0.0001, 10 cm vs tumor; ^***^ *p* = 0.031 10 cm vs tumor.

**Table 1B:** Analysis of differences in KLK6 staining based on the status of *K-RAS* oncogene (Wilcoxon rank-sum test)

Distance from tumor site	Localization	K-RAS status	mean	median	*p*-value
		K-RAS mutant	66.0 (54.1)	90	
10 cm	Crypts	K-RAS wild	0.(0)	0	0.0515
	Luminal	K-RAS mutant	136.0 (97.1)	100	
	surface	K-RAS wild	33.0 18.8)	30	0.0445
		K-RAS mutant	46.0 (58.1)	30	
1 cm	Crypts	K-RAS wild	12.0 (13.3)	10	0.4685
	Luminal	K-RAS mutant	106.0 (78.09)	100	
	surface	K-RAS wild	41.0 (56.6)	25	0.1249
		K-RAS mutant	139.0 (65.7)	100	
tumor	Crypts	K-RAS wild	113.0 (84.5)	130	0.8387
	Luminal	K-RAS mutant	36.0 (49.7)	0	
	surface	K-RAS wild	0 (0)	0	0.2126

**Figure 5:**
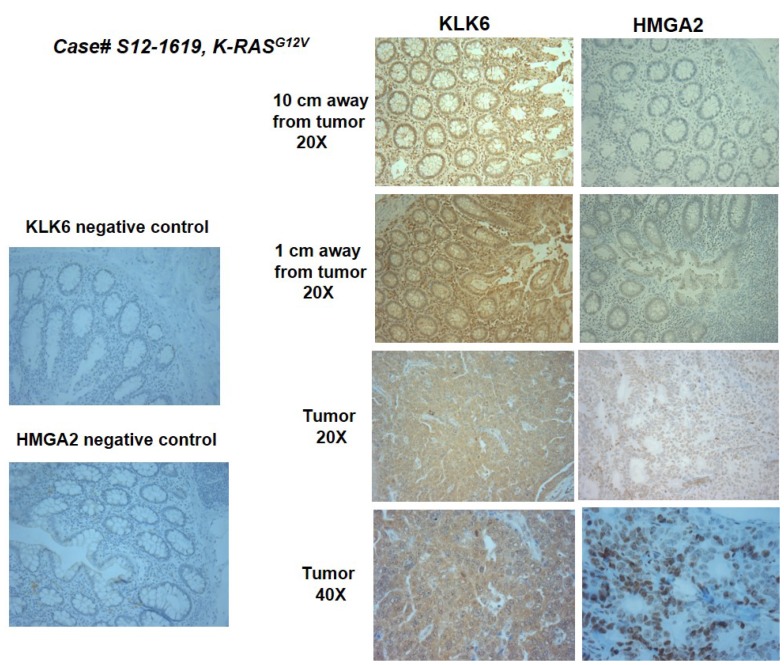
Representative images of KLK6 and HMGA2 IHC staining in the surgical material of a colon cancer patient. Staining was done in a morphologically normal tissue collected 10 cm away of a tumor, tissue collected 1 cm away from a tumor site and in a tumor sample.

We further performed IHC staining for the HMGA2 protein in the subset of these clinical samples. The subset selected for analysis included patients that tested negative (*N* = 4) and positive (*N* = 3) for the *K-RAS* mutation. Tumor, paired adjacent, and apparently normal tissue blocks were sectioned sequentially and processed for KLK6 and HMGA2 staining. Staining patterns for KLK6 and HMGA2 expression were scored by a blinded pathologist. Nuclear HMGA2 staining was observed in the deeply invasive tumors and adjacent tissue samples, which scored high for KLK6 (Figure 5 and Supplementary Table 1). Comparison of the staining intensity between KLK6 and HMGA2 demonstrated a Spearman correlation of 0.70 (*p* < 0.001) with significant positivity in tumor (0.91, *p* < 0.01) and adjacent (0.81, *p =* 0.003) tissues (Table 2A–2E). No correlation between KLK6 and HMGA2 staining was found in the morphologically normal tissue samples. Supplementary Table 1 presents some pathological characteristics of the analyzed cases, including the disease stage, lymph node positivity for tumor cells, and disease recurrence within 5 years after surgery. All seven cases used for the analysis of KLK6 and HMGA2 expression were diagnosed as moderately differentiated adenocarcinomas. In this limited set, the disease recurrence was observed in patients who had high KLK6 score and positive HMGA2 staining in the tumor samples (Supplementary Table 1).

**Table 2A:** Correlation analysis of KLK6 and HMGA2 levels in CRC samples (Spearman correlation analysis)

Tissue Type	Spearman correlation coefficient
All (*N* = 21)	0.70; *p* < 0.001
Tumor (*N* = 7)	0.91; *p* < 0.01
1 cm away from tumor (*N* = 7)	0.81; *p* = 0.03
10 cm away from tumor (*N* = 7)	NA

**Table 2B:** Agreement between KLK6 (>0) and HMGA2 (>0)

All	HMGA2=0	HMGA2>0
**KLK6=0**	6	0
**KLK6>0**	9	6

McNemar’s test: *p <* 0.01; kappa=0.28 (95% CI: 0.03, 0.52).

**Table 2C:** Agreement between KLK6 (>0) and HMGA2 (>0)

Tumor	HMGA2=0	HMGA2>0
**KLK6=0**	0	0
**KLK6>0**	4	3

McNemar’s test: NA; kappa=NA.

**Table 2D:** Agreement between KLK6 (>0) and HMGA2 (>0)

Adjacent	HMGA2=0	HMGA2>0
**KLK6=0**	2	0
**KLK6>0**	2	3

McNemar’s test: *p =* 0.16; kappa=0.46 (95% CI: -0.07, 0.99).

**Table 2E:** Agreement between KLK6 (>0) and HMGA2 (>0)

Normal	HMGA2=0	HMGA2>0
**KLK6=0**	4	0
**KLK6>0**	3	0

McNemar’s test: NA; kappa=NA.

### Analysis of KLK6, HMGA2 and LIN28B expression in CRC tumors from The Cancer Genome Atlas (TCGA) database

**Figure 6:**
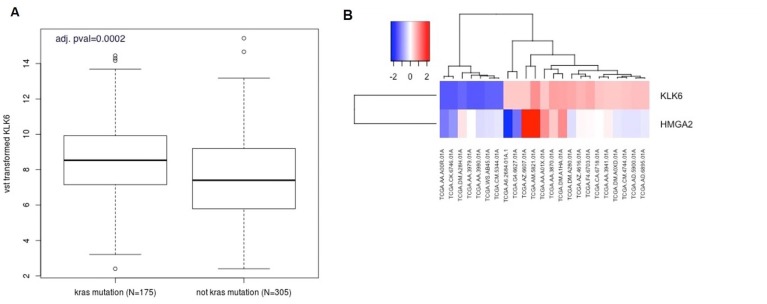
Bioinformatic analysis of KLK6 and HMGA2 expression in the CRC patients. (**A**) Boxplot analysis of KLK6 expression in K-RAS–mutant and non-K-RAS mutant samples in colon adenocarcinomas from the TCGA database. (**B**) Analysis of KLK6 and HMGA2 expression in 23 TCGA CRC samples stratified by high and low expression of KLK6. Heatmap shows the correlation of KLK6 with HMGA2 expression in these samples. *P =* 0.02, *r* = 0.47 by Spearman correlation analysis.

We analyzed KLK6 expression in *K-RAS* mutant samples and non-*K-RAS* mutant samples in colon adenocarcinomas from the TCGA database (https://gdc.cancer.gov/). Raw RNAseq counts were normalized and samples were grouped by the presence or absence of *K-RAS* mutations. We found that the level of KLK6 expression was overall higher in *K-RAS* mutant samples than in non-*K-RAS* mutant samples (*p =* 0.0002) (Figure 6A).

We also stratified the patients’ samples by top outliers in KLK6 high and low expression and performed Spearman correlation analysis of HMGA2, LIN28B and KLK6. In this group of 23 samples we found a correlation between HMGA2 and KLK6 expression (Figure 6B, *r* = 0.47, *p =* 0.02). LIN28B expression did not significantly correlate with KLK6 expression in this set. TCGA colon cancer database contains the samples of early colon cancer stages, therefore, the performed analysis should not be interpreted as characteristics of advanced colon tumors.

## DISCUSSION

Approximately 50% of CRC patients with tumor detected in the lymph nodes (stage III CRC) and 25% of patients without lymph node involvement (or stage I and II) will undergo recurrence [27]. The lymph node analysis of KLK6 mRNA in the CRC patients showed that presence of KLK6 in lymph nodes directly relates to the risk of disease recurrence and that patients with the highest KLK6 levels in their lymph nodes have the shortest survival time [28].

To understand how KLK6 contributes to the poor prognosis in the CRC patients we stably expressed enzymatically active and inactive KLK6 in Caco-2 colon cancer cells. The Caco-2 cell line carries a wild-type *K-RAS* oncogene, expresses a very low basal level of KLK6 and exhibits a low tumorigenicity *in vivo*, unless the mutant *K-RAS* is introduced into this cell line [7]. Therefore, the Caco-2 cell line presents an ideal model system for testing the phenotypic and molecular consequences of KLK6 overexpression. Using the Caco-KLK6 model and the previously characterized HCT116-shKLK6 cell model [17] we showed that the proteolytic activity of the KLK6 enzyme is directly responsible for the invasive properties of colon cancer cells. To investigate the mechanism of KLK6-mediated invasion in more detail we evaluated the status of the reported earlier putative mediators of cell invasion, such as TGF-β2 ligand, EMT markers, and regulators of transcription [17].

TGF-β2 belongs to a superfamily of polypeptide growth factors and can act as both a tumor suppressor, by inhibiting epithelial cells growth, and as a promoter of tumor progression and metastasis [29]. TGF-β2 is considered to be a hallmark of various malignant tumors, including pancreatic carcinoma, glioma, melanoma and colorectal cancer as it is implicated in regulation of multiple molecular pathways [30]. TGF-β2 is synthesized as a propeptide precursor containing a prodomain, latency-associated peptide (LAP) complex and the mature domain, similarly to other TGF-β isoforms, and is secreted in an inactive form (reviewed in [31]). We detected the active TGF-β2 protein in the conditioned media of Caco-KLK6 clones and shKLK6 clone 3, only when the enzymatically active KLK6 was expressed. This finding suggests that the KLK6 protease may be involved in the TGF-β2 ligand activation. The current observation of the KLK6-mediated TGF-β2 expression corroborates with our earlier computational predictions of KLK6 and TGF-β interactions [32].

Furthermore, we observed the elevated levels of phosphorylated SMAD2/3 receptors, upon overexpression of enzymatically active KLK6 in Caco-2 colon cell line, which demonstrates that the KLK6 enzyme activates SMAD signaling. Although HCT116 cells have a truncated mutation of type II TGF-β receptor (TGFR2), SMAD2 protein was phosphorylated in the shKLK6 isogenic clone upon re-expression of the enzymatically active KLK6, because the TGFR2 protein retains its function in this cell line as well as other CRC with microsatellite instability [33].

The TGF-β/SMAD signaling pathway is a major inducer of the EMT [34]. During neoplastic growth and progression, cells, which undergo EMT, acquire resistance to apoptosis, stress, inhibition of senescence, and invasive and metastatic properties. We evaluated the status of the epithelial cell marker E-cadherin and the mesenchymal markers involved in cytoskeleton reorganization, i.e. vimentin, ZO-1, Snail, Slug 1, ZEB1, all of which are known hallmarks of the EMT [18]. In the Caco-KLK6 model, the levels of E-cadherin and Snail proteins did not change. At the same time, expression of β-catenin was induced 2-8-fold in the enzymatically active Caco-KLK6 clones, which suggests that β-catenin may coordinate the EMT in Caco-2-KLK6 model. In HCT116-shKLK6 cells, β-catenin expression was not altered, because in this cell line β-catenin is mutated [35]. Besides the known function of β–catenin in regulation of the Wnt signaling pathway [36], it can also be involved in the regulation of cell invasion through its complex with cadherins [37]. Future evaluation of β-catenin/E-cadherin complex and/or its translocation to the nucleus will help us to determine the exact function of β-catenin in KLK6 overexpressing colon cancer cells. Snail is a major transcription factor, which downregulates expression of other epithelial molecules during transition to the mesenchymal phenotype and has been associated with increase in tumor grade, metastasis, recurrence, and poor prognosis [38]. In shKLK6 cells, the intracellular level of Snail was suppressed two-fold and ZO-1 protein was suppressed 3-4-fold, whereas E-cadherin was elevated two-fold. These changes suggest the reversal of the EMT in the HCT116 cell line with knocked down of KLK6 expression**.** It is important to note, that although we reported here KLK6-dependent alterations in SMAD signaling and EMT via TGF-β2 ligand, other possible inducers of invasion are Notch and Nodal ligands [39]. Contributions of the KLK6 enzyme into these pathways will be important to evaluate in the future.

The HMGA2 transcript level was significantly elevated in the Caco-KLK6 cell model (5–10 times compared to Mock cells). On the contrary, expression of the HMGA2 transcription factor was inhibited by 2-5-times in HCT116-KLK6 knockdown clones. HMGA2 is involved in neoplastic transformation and its expression is associated with poor survival in colorectal cancer [21]. Some findings also implicate HMGA2 in the EMT program downstream of TGF-β [25, 40]. HMGA2 mRNA also is the established target of a tumor suppressor let-7 miRNA family [41]. The members of the let-7 family of miRNA have been reported as putative regulators of KLK6 expression [42, 43]. Based on the published data and our current finding, the let-7 family of miRNAs may control the expression of both HMGA2 and KLK6. The let-7, in turn, can be regulated by LIN28 ribosomal binding protein through its direct binding to either precursor let-7 (pre-let-7) or primary (pri-let-7) miRNAs [44, 45]. Two LIN28 paralogs, LIN28A and LIN28B, have been reported in mammalian cells, and they can regulate let-7 biogenesis through different molecular pathways [46]. We evaluated the status of LIN28 proteins in our cell models. The LIN28A protein was not altered by KLK6 in our cell models, but the intracellular level of LIN28B was found to be elevated in KLK6-overexpressing Caco-2 isogenic lines and was suppressed in HCT116 isogenic clones upon KLK6 knockdown. Although the LIN28B overexpression in colon cancer has been linked to colon cancer progression [47], the molecular pathways connecting LIN28B and other proteins directly involved in invasion have not been identified. Therefore, our findings suggest further investigation of the relationship between LIN28B and KLK6 expression. It is important to note that expression patterns of both HMGA2 and LIN28B correlated with KLK6 expression but not with the proteolytic function of KLK6.

A limited analysis of the expression and secretion patterns of KLK6 in CRC patients, point to the individual variations in KLK6 expression and suggest that further stratification of patients is needed when evaluating KLK6 in colon cancer [9, 12]. Our analysis of KLK6 expression in CRC patients by IHC shows the statistically significant increase in the staining score for KLK6 protein in the morphologically normal tissue 10 cm away from tumors, which were tested positive for K-RAS mutation (*p =* 0.05 for crypts staining and *p =* 0.0445 for luminal surface area). The K-RAS oncogene is mutated in about one-quarter of advanced colon adenomas [48] and in nearly one-half of colon cancers and have been linked to the lower overall patients’ survival due to lack of effective treatment options [49].

In the analyzed subset of clinical samples, a positive correlation between KLK6 and HMGA2 was found in the tumor samples and adjacent tissues (Spearman correlation coefficient is 0.91, *p* < 0.01, and 0.81, *p =* 0.03, respectively). Moreover, disease recurrence was noted in patients with high KLK6 scores and positive HMGA2 staining. Although more robust analysis of the clinical cases is required, our current observations suggest that KLK6 may contribute to the LIN28B-let7-HMGA2 axis and may serve as an early marker of disease recurrence.

Overall, we determined that enzymatic activity of KLK6 promotes the mesenchymal phenotype in colon cells via induction of the EMT pathway and that KLK6 overexpression correlates with expression of regulatory protein HMGA2.

## MATERIALS AND METHODS

### Cell lines

Caco-2 and HCT116 isogenic clones were used in this study. Molecular features of these cell lines are presented in Supplementary Table 2. HCT116 cell model. HCT116 negative control cells and cells with knockdown expression of *KLK6* (Control clone 1 and shKLK6 cell lines) have been previously described [17]. Control clone 1 and shKLK6 isogenic clones 2–4 were maintained in Dulbecco’s Modified Eagle Medium (DMEM) with 4.5 mg/L glucose, L-glutamine w/o sodium pyruvate, supplemented with 10% FBS and 1% penicillin/streptomycin with addition of selection marker puromycin at the concentration of 0.5 μg/mL of media (Thermo Fisher Scientific Inc). Caco-2 cell model. Caco-2 parental cells were transfected using LipofectAMINE-3000 with a pcDNA3.1(+) plasmid driving the expression of the wild-type prepro*KLK6* or the mutant prepro*KLK6* (S197A) under a cytomegalovirus (CMV) promoter [11], according to the manufacturer’s instructions (Life Technologies, Inc.). LipofectAMINE-3000 was removed after 24 hours, complete medium was added for another 24 hours and then was replaced with selection medium containing 350 μg/mL of a selection marker G418. Individual clones of stably-transfected cells were picked after six weeks of growth in the selection media. Caco-2 isogenic clones were maintained in Modified Eagle Medium (MEM) (Thermo Fisher Scientific, Inc.) with 4.5 mg/L glucose, L-glutamine and supplemented with 10% FBS, 1% penicillin/streptomycin and 350 μg/mL of G418.

### Cell growth

Caco-2 parental cells, and clonally collected cells expressing pcDNA3.1 empty plasmid, Caco-2 Mock transfected clone #10 (named Mock), expressing enzymatically active KLK6, Caco-KLK6 wt #5 clone (KLK6 wt) and a clone expressing enzymatically inactive KLK6, Caco-KLK6 S197A clone #5 (KLK6S197A), were plated in 6 well plates at a concentration of 0.6 × 10^6^ cells/well and incubated for 37º C in 5% CO_2_. All cells were plated in triplicate. The cells were grown for 7 days. Cells were harvested and viable cell number were determined using the trypan blue dye exclusion method on a Vi-Cell Series Cell Viability Analyzer (Beckman Coulter Inc.). Determination of population doubling time was performed by plotting viable cell number *vs* days after subculture for the logarithmic phase of growth. Results and corresponding SDs were derived from three independent experiments.

### Caco-KLK6 cell migration/invasion assay

Caco-2 Mock and Caco-2-KLK6 isogenic clones KLK6 wt 5 and KLK6 S197A 5 were seeded in 24-well chambers with either control plastic inserts or Matrigel coated inserts with a 0.8 μm pore size (Corning Inc., Corning, NY, USA). In the lower chamber of each well 0.5 mL of media with 10% FBS was added as a chemoattractant. Cell suspension was prepared at a concentration of 0.5 × 10^6^ cells/ml in serum-free medium. 200 μL of cell suspensions (1 × 10^5^ cells) were plated into inserts. The cells were allowed to migrate/invade for 48 hours. The media was discarded and inserts were rinsed briefly with 1X PBS and swabbed gently with a cotton tip inside inserts to remove non-migrating or non-invading cells. Subsequently, migrating/invading cells were fixed in 100% methanol for 2 minutes, stained with 1% toluidine blue in 1% borax) for 2 minutes, rinsed twice in ddH_2_O, swabbed gently again inside with a cotton tip and air-dried. Control inserts were included following similar treatment. The stained membranes were cut from the inserts and incubated in 200 μl of 0.1 M citric acid in a 96 well plate while shaking for 5 minutes on a high-speed titer plate shaker. The solution was transferred to a new designated well in a new 96 well plate and read at 560 nm on a Synergy 2 Multi-Detection Microplate Reader (Bio-Tek Instruments, Inc.,Winooski, VT, USA). Each migration/invasion experiment was carried out in sextuplet and was repeated twice.

### Transient transfection with KLK6 plasmids for matrigel invasion assay

Control clone 1 and shKLK6 clone 3 were seeded in 100 mm^2^ Petri dishes at the concentration of 1.2 × 10^6^ cells. Twenty-four hours after subculture Control clone 1 was transfected with an empty pcDNA3.1 vector and shKLK6 clone 3 cells were transfected with pcDNA3.1 vector or KLK6 wt and KLK6 S197A plasmids using the LipofectAMINE 2000 reagent according to the manufacturer’s instructions. Twenty-four hours after transfection (48 hours after subculture) transfected cells and not transfected Control clone 1 cells were trypsinized and seeded into the Matrigel invasion chambers. Matrigel invasion assays were carried out as described elsewhere [17]. Cells were allowed to invade for another 48 hours and processed for colorimetric assay.

### RNA isolation

Total RNA extraction from colon cancer cell lines was done 48 hours after subculture using the miRVana miRNA isolation kit (Ambion™, Thermo Fisher Scientific, Inc.). RNA purity was assessed using a Nanodrop spectrophotometer (ND-2000; Thermo Fisher Scientific, Inc.). All RNA samples prepared for experiments had 260/280 absorption values between 1.8–2.0.

### Quantitative real-time PCR

Total RNA was isolated as described above. Reverse transcription to produce cDNA template was completed using the Applied Biosystems High Capacity cDNA Reverse Transcription Kit (Part #4368814). qPCR was performed using TaqMan^®^ probes (Applied Biosystems, Thermo Fisher Scientific, Inc.) specific for the mRNAs of interest: KLK6 (Hs00160519_ml), TGF-β2 (Hs00234244_ml), HMGA2 (Hs04397751_ml). 0.2 μg of total RNA was reversed transcribed into cDNA in a 20 μL reaction with random hexamers under thermal condition recommended by the protocol. Real-time PCR amplification was performed with the ABI PRISM 7700 SDS instrument (Applied Biosystems, Life Technologies, Inc.), under the universal thermal cycling conditions recommended by the Assay-on-Demand products protocol. Negative controls without template were included in each plate to monitor potential PCR contamination. The expression of genes was tested in triplicate and each reaction was run in duplicate. To determine the relative expression level of each target gene, the comparative *CT* method was used. The *CT* value of the target gene was normalized by the endogenous reference β2-microglobin (β2M, FAM (Hs99999907_m1)). The relative expression of each target gene was calculated via the equation 2^-Δ^ *C_T_* where Δ*C_T_* = *C_T_*_(target)_ – *C_T_*_(endogenous control)_.

### Western blot analysis

Western blot analyses were performed as previously described [17]. The primary and secondary antibodies used in this study and blotting conditions are presented in Supplementary Table 3. Proteins of interest were detected with an enhanced chemiluminescent detection reagent (Thermo Fisher Scientific Super Signal West Pico or West Femto Chemiluminescent Substrate, Cat#34077 or 34095, respectively) and exposed to film (Denville Scientific Inc., HyBlot CL, Autoradiography film, Cat#E3012).

All western blots were repeated at least twice. Image processing program, Image J was used for evaluation of Western blot bands intensity where specified (Image J, http://rsb.info.nih.gov/ij/docs/menus/analyze.html#gels). Protein bands of interest were normalized to β-actin or total proteins for phosphorylated state.

### Human tissue samples

De-identified human tissue samples were obtained from the University of Arizona Cancer Center Biospecimen Repository, which is maintained by the Tissue Acquisition and Cellular/Molecular Analysis Shared Resource (TACMASR). Tissues were collected under the University of Arizona Internal Review Board (IRB)-approved protocol #09-0217-04). Set of primary tumor and matching adjacent and normal tissues (1 cm and 10 cm away from the tumor, respectively) from 10 CRC patients was analyzed for KLK6 and HMGA2 expression by immunohistochemistry (IHC). Patients’ consent for tissue collection and use was obtained before surgery. Samples were de-identified prior to release and were verified based on patients’ pathology reports and evaluation of H&E-stained slides by a certified pathologist.

### KLK6 and HMGA2 immunohistochemistry

Routine hematoxylin and eosin (H&E) stains were performed on 3-micron sections of tissue cut from the formalin fixed, paraffin embedded (FFPE) blocks. Detection of primary antibodies was performed on a Discovery XT Automated Immunostainer (Ventana Medical Systems, Inc., Tucson, AZ, USA). KLK6 Immunohistochemistry (IHC) was performed using a goat Kallikrein 6 polyclonal antibody (R&D Systems, Minneapolis, MN, USA, Cat. #AF2008, dilution 1:150) and a biotinylated anti-goat IgG (H&L) Vector Laboratories Inc., Burlingame, CA, dilution of 1:200). HMGA2 IHC was done using rabbit HMGA2 antibody (Cell Signaling Technology, Inc., Danvers, MA, USA, dilution 1:100) and a biotinylated anti-rabbit IgG ((H&L) Vector Laboratories Inc., Burlingame, CA, USA, dilution 1:200).

De-paraffinization and cell conditioning (antigen retrieval) using a borate-EDTA buffer at 100°C were performed online using VMSI validated reagents. Detection of primary antibodies was accomplished using a biotinylated-streptavidin-HRP and DAB system. Following staining on the instrument, slides were dehydrated through graded alcohols to xylene and coverslipped with Pro-Tex mounting medium. All slides were analyzed by an experienced pathologist with over 30 years of experience, who was blinded to treatment categories. Results are presented as a long score based on the sum of intensity of staining multiplied by the percent of stained tissue area. The rating scale of 0 to 4 was used with the following scoring criteria: 0, no staining; 1+, weak diffuse staining (may contain stronger intensity in <10% of the cells); 2+, moderate strong staining in 10%–90% of the cells, and 3+ strong intense staining, more than 90% of the cells stained with strong intensity. Staining was analysed in three fields per slide. KLK6 staining was analyzed in 10 patients, HMGA2 staining was analyzed in 7 patients.

### Enzyme-linked immunosorbent assay (ELISA)

*KLK6 ELISA*. An ELISA kit for the detection of human KLK6 was obtained from Boster Immunoleader (Pleasanton, CA, USA). The assay was performed according to manufacturer’s protocol. Conditioned media from all tested cell lines was collected at 48 hours after subculture. KLK6 levels are expressed as picograms per milliliter of media. The plate was read at 490 nm within 30 min of assay ending on a Synergy 2 Multi-Detection Microplate Reader (Bio-Tek Instruments, Inc., Winooski, VT, USA).

*TGF-β2 ELISA*. For TGF-β2 analysis cells were seeded on 100 mm plates in serum-free media (Caco-KLK6 clones) or in Matrigel invasion chambers (transiently transfected shKLK6 cells) and conditioned media was collected 48 hours after subculture. Active TGF-β2 level in the conditioned media was determined using Quantikine ELISA for human TGF-β2 immunoassay (R&D Systems, USA) according to the manufacturer’s instructions, but without pre-treatment with 20 μl of 1M HCl, step which is used to activate latent TGF-β2. Samples were pipetted into microplate wells pre-coated with a monoclonal antibody specific for TGF-β2 and incubated for 2 hours at room temperature. An enzyme-linked polyclonal antibody specific for TGF-β2 was then added and the color intensity was determined using a Synergy 2 Multi-Detection Microplate Reader (Bio-Tek Instruments, Inc., Winooski, VT, USA). Media from Caco-KLK6 cell model (Mock, KLK6 wt5 and KLK6S197A5 clones) and shKLK6 3 clone transiently transfected with pcDNA 3.1, KLK6 wt and KLK6S197A plasmids were tested. Levels of TGF-β2 ligand were expressed as picograms of protein per milliliter of media.

### SCID mouse model

*In vivo* studies were conducted on 6-7 week-old male and female Severe Combined Immunodeficient (SCID) mice (CB-17/IcrACCscid) originally purchased from Taconic Inc. (Germantown, NY, USA). The SCID mouse colony is maintained by the University of Arizona Cancer Center Experimental Mouse Shared Resource (EMSR) and housed at animal facility in the University of Arizona Cancer Center. The facility is AAALAC accredited, and holds an Animal Welfare Assurance # with the Public Health (File#A-3248-01). Mouse colonies were maintained under microisolation and were handled and manipulated only in laminar flow hoods. All procedures involving animals were conducted according to the University of Arizona Animal care and Use Committee policies.

For the animal survival study, Caco-2 parental cells and Caco-2 isogenic clonally selected cell lines Mock, KLK6 wt 5 and KLK6 S197A 5 were injected between the mucosa and the muscularis externa layers of the cecal wall of anesthetized mice and the concentration of 3 × 10^6^ cells in 30 μL of saline solution (10 animals per experimental group) using a 30-gauge needle. Animals were observed twice weekly for adverse signs associated with tumor growth (e.g., >20% body weight loss, immobility, loss of grooming), at which time mice were euthanized. Metastatic colonization and tumors formed were evaluated postmortem.

### Statistics

Statistical analysis of cell culture experimental data was performed using paired *t-*test or the analysis of variance (ANOVA) (Microsoft Excel, Microsoft Corp.). A nonparametric test (Wilcoxon rank test) was performed for the analysis of KLK6 gene expression in the CRC samples. The Wilcoxon signed rank test was used to test the difference between the KLK6 staining in the CRC samples. For the positive KLK6 staining (staining score >0) versus negative staining (score = 0), the Fisher’s exact test was employed. The Tukey adjustment was applied for multiple comparisons. For correlation analysis of KLK6 and HMGA2 levels in CRC samples Spearman test based on the rank of staining score and agreement analysis based on negative or positive (i.e. >0) staining were used. The Kaplan–Meier method was used for survival analysis. The long-rank test was employed to test the difference in the animal survival curves. Fisher’s exact test was employed to test the difference in incidence of metastases between genotype groups.

### Transcriptome data analysis from TCGA

Raw RNA-Seq counts were downloaded from Genomics data commons data portal using GDC API. The data was normalized using Variance Stabilizing Transformation [50] and transformed data was utilized for further analysis. All analyses and plots were generated using R (v3.4.3).

## SUPPLEMENTARY MATERIALS



## Abbreviations

KLK6: Kallikrein 6
CRC: colorectal cancer
EMT: epithelial-mesenchymal transition
ERK: extracellular signal-regulated kin ases
IHC: immunohistochemistry
qPCR: quantitative real time PCR
β2M: β2-microglobin
GAPDH: glyceraldehyde-3′-phosphate dehydrogenase
HMGA2: high-mobility group AT-hook 2
SMAD2: Mothers Against DPP Homolog 2
RalA: RAS-like small GTPase A
MAPKs: mitogen-activated protein kinases
TGF-β2: transforming growth factor beta 2
AKT/PKB: v-Akt Murine Thymoma Viral Oncogene Protein Kinase B
