# Supplementary material for: Kallikrein 6 protease advances colon tumorigenesis via induction of the high mobility group A2 protein
Source: Oncotarget. 2019 Oct 22;10(58):6062–78. doi: 10.18632/oncotarget.27153 (PMC6817440; doi:10.18632/oncotarget.27153)
Supplement: Supplementary file 1 [file oncotarget-10-6062-s001.pdf]

Kallikrein 6 protease advances colon tumorigenesis via induction of the high mobility group A2 protein

SUPPLEMENTARY MATERIALS

Supplementary methods

Subcutaneous SCID mouse xenograft model

For the tumorigenesis study, HCT116 parental (5 × 10^6 or 10 × 10^6 cells/100 µL saline/mouse) and Caco-2 parental cell lines (10 × 10^6 cells/100 µL saline /mouse) were injected subcutaneously into the flank area of SCID mice. Six mice per group were tested. The body weight of the mice and the tumor size were observed and recorded twice per week. Tumor volume was determined in mm^3 by using the formula ($a^2 \times b/2$), where a is the smaller of the measurements to estimate size. The maximum tumor volume permitted was 1,000 mm^3.

Transient Transfection with KLK6 plasmids for Western blot analysis of SMAD proteins

HCT116 isogenic clones shKLK6 2, 3 and 4 were seeded in 100 mm dishes at 2 × 10^6 cells. Cells were transfected with LipofectAMINE 3000 reagent according to the manufacturer's instructions. Cells were harvested 48 hours and processed for Western blot analysis.

Supplementary Data

KLK6 expression correlates with tumorigenic potential of colon cell lines

We compared KLK6 expression and secretion in colon cell lines, Caco-2 (wild type K-RAS) and HCT116 (*K-RASG*^{12D}). Supplementary Figure 1A–1C demonstrate that HCT116 cells express and secrete significant amounts of KLK6 compared to Caco-2 cells. We also tested the ability of Caco-2 and HCT116 cell lines to form xenograft tumors in SCID mice. Caco-2 cells (10 × 10^6 cells/ml) and HCT116 cells (5 or 10 × 10^6 cells/ml) were injected subcutaneously into the flank area of SCID mice ($N = 6$ animals per group) and the tumor growth was monitored for twenty days. During the observed period, Caco-2 injected animals did not develop any tumors, but animals injected with HCT116 cells formed subcutaneous tumors whose volume positively correlated with the number of injected cells. Specifically, at the time of sacrifice, animals injected with 5 × 10^6 cells/animal developed tumors with approximately 400 mm^3 average volume, whereas animals injected with at 10 × 10^6 cells/animal had approximate tumor volume of 1000 mm^3 (Supplementary Figure 1D).

Supplementary Table 1: Characteristics of surgical cases used for KLK6 and HMGA2 IHC analysis

Case #	K-RAS mutation	Disease stage	Lymph node positive for metastasis/ analyzed	Tumor IHC score in		Recurrence
				KLK6	HMGA2	
**S08-4669**	No	p3N1M1	1/12	250	neg	Unrelated
**S09-6781**	No	pT3N1Mx	1/10	200	neg	Unrelated
**S08-8906**	No	pT3N1Mx	2/13	200	neg	No recurrence
**S10-10827**	No	p3N1M1	2/14	300	180	Metastatic adenocarcinoma
**S11-2111**	G13D	pT4bN0Mx	0/9	300	200	Metastatic adenocarcinoma
**S11-12211**	G13D	pT4aN2a	4/12	300	200	Metastatic adenocarcinoma
**S12-1619**	G12V	pT4aN2b	7/8	400	200	Metastatic adenocarcinoma

Supplementary Table 2: Molecular characteristics of Caco-2 and HCT116 colon cell lines used in this study

Cell Line (ATCC)	Origin	Epigenetic and genetic features	Genetic phenotype
Caco-2 (HTB-37™)[1]	Colorectal adenocarcinoma	KRAS: Wild type (wt)<br>APC: Mutation in codon 1367 (CAG-TAG (stop))<br>p53: Mutation in codon 204 (E204X)<br>PTEN: wt<br>PIK3 CA: wt<br>β-catenin: Mutation in codon 245 (GGC-GCC (missense) exon 5)<br>SMAD4 (D351H)	Microsatellite stable (MSS)
HCT116 (CCL-247™)[2]	Colorectal carcinoma	K-RAS: Mutation in codon 13 (G13D)<br>APC: Wild-ype (wt)<br>p53: wt<br>PTEN: wt<br>TGFβRII: Frameshift Mutation<br>PIK3 CA: Mutation in codon 1047 (H1047R)<br>βcatenin: Mutated codon 45 (3 base deletion exon 3)	Microsatellite Instability (MSI)

[1]https://www.atcc.org/Products/All/HTB-37.aspx#generalinformation
[2]https://www.atcc.org/products/all/CCL-247.aspx

Supplementary Table 3: List of antibodies used in this study

Antibody	Manufacturer (City, State)	Primary antibody & dilution Factor	Secondary species & dilution factor	Primary antibody & dilution media
Anti-β-actin	Sigma-Aldrich (St. Louis, MO)	#A5441 Clone AC-15 1:5000	Goat Anti-mouse IgG Invitrogen #626520 1:10,000	1.5% BSA Shaking 1 h RT
Human KLK6/Neurosin	R&D (Pleasanton, CA)	#AF2008 1:100	Donkey Anti-goat IgG Santa Cruz #2020 1:2000	5% BSA 4ºC O/N shaking
LIN28A	Cell Signaling	#8641 1:1000	Anti-rabbit IgG #7074 1:2000	5% BSA 4ºC O/N shaking
LIN28B	Cell Signaling	#4196 1:1000	Anti-rabbit IgG #7074 1:2000	5% BSA 4ºC O/N shaking
p-SMAD2 (Ser 465/467)	Cell Signaling	#3108 1:1000	Anti-rabbit IgG #7074 1:2000 1:2000	5% BSA 4ºC O/N shaking
p-SMAD 3 (Ser423/425)	Cell Signaling	#9520 1:1000	Anti-rabbit IgG #7074 1:2000	5% BSA 4ºC O/N shaking
SMAD2	Cell Signaling	#5339 1:1000	Anti-rabbit IgG #7074 1:2000	5% Blotto A 4ºC O/N shaking
SMAD3	Cell Signaling	# 9523 1:1000	Anti-rabbit IgG #7074 1:2000	5% Blotto A 4ºC O/N shaking
SMAD4	Cell Signaling	#38454 1:1000	Anti-rabbit IgG #7074 1:2000	5% Blotto A 4ºC O/N shaking
Vimentin	Cell Signaling	#5741 1:1000	Anti-rabbit IgG #7074 1:2000	5% Blotto A 4ºC O/N shaking
N-cadherin	Cell Signaling	#13116 1:1000	Anti-rabbit IgG #7074 1:2000	5% Blotto A 4ºC O/N shaking
β-catenin	Cell Signaling	#8480 1:1000	Anti-rabbit IgG #7074 1:2000	5% Blotto A 4ºC O/N shaking
ZO-1	Cell Signaling	#8193 1:1000	Anti-rabbit IgG #7074 1:2000	5% Blotto A 4ºC O/N shaking
E-cadherin	Cell Signaling	#3195 1:1000	Anti-rabbit IgG #7074 1:2000	5% Blotto A 4ºC O/N shaking
Snail	Cell Signaling	#3879 1:1000	Anti-rabbit IgG #7074 1:2000	5% Blotto A 4ºC O/N shaking
Slug	Cell Signaling	#9585 1:1000	Anti-rabbit IgG #7074 1:2000	5% Blotto A 4ºC O/N shaking
ZEB1	Cell Signaling	#3396 1:1000	Anti-rabbit IgG #7074 1:2000	5% Blotto A 4ºC O/N shaking

RT: Room temperature; O/N: Overnight

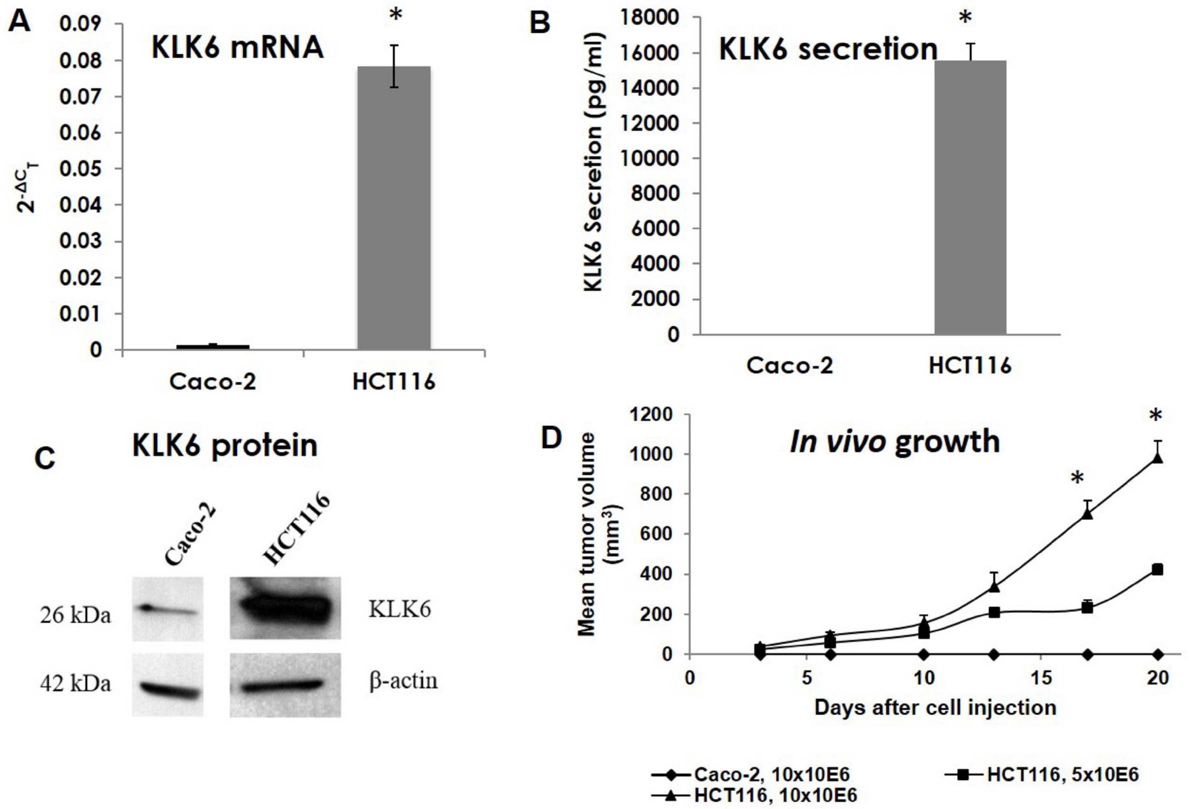

Supplementary Figure 1: Analysis of KLK6 expression and secretion in Caco-2 and HCT116 colon cancer cell lines *in vitro* and their tumorigenicity *in vivo*. (**A**) KLK6 mRNA levels in cells 48 hours after subculture by qPCR. (**B**) Levels of secreted KLK6 protein in conditioned media 48 hours after subculture. (**C**) Western blot analysis of the intracellular level of KLK6 48 hours after subculture. (**D**) Tumor growth rates of Caco-2 and HCT116 cells in SCID mouse xenografts. Caco-2 cells were injected at 10×10^6 cells per 100 µL PBS and HCT116 were injected at 5×10^6 cells per 100 µl PBS and 10×10^6 cells per 100 µL PBS into the two lower flanks of each mouse. Tumor growth was measured twice a week and calculated as described in Supplementary Methods section. Data presented as average tumor volume + standard error. $^*p < 0.05$. *P* values were calculated by paired *t*-test (A–C) or ANOVA (D). (A–C) are representative of three independent experiments performed with triplicates. Tumor growth analysis (D) is a representative two independent experiments.

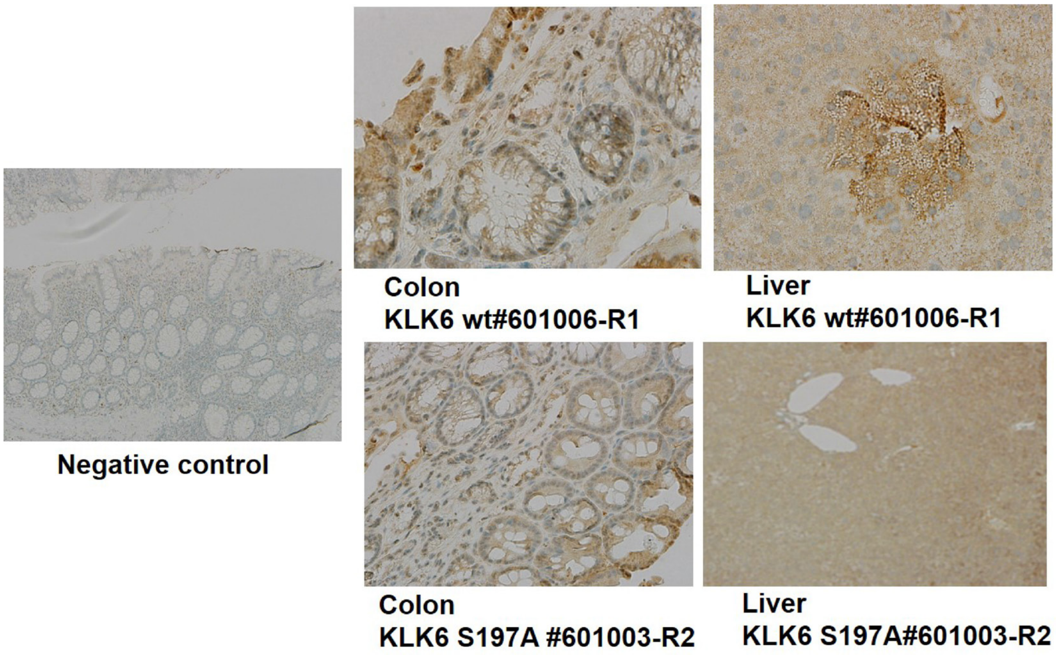

Supplementary Figure 2: Analysis of KLK6 expression in Caco-KLK6 orthotopic colon cancer model by IHC. Representative images of KLK6 staining in the colon tumor and the liver of tumor-bearing animals injected with KLK6 wt cells (animal # 601006-R1) and KLK6S197A cells (animal # 601003-R2) are shown.

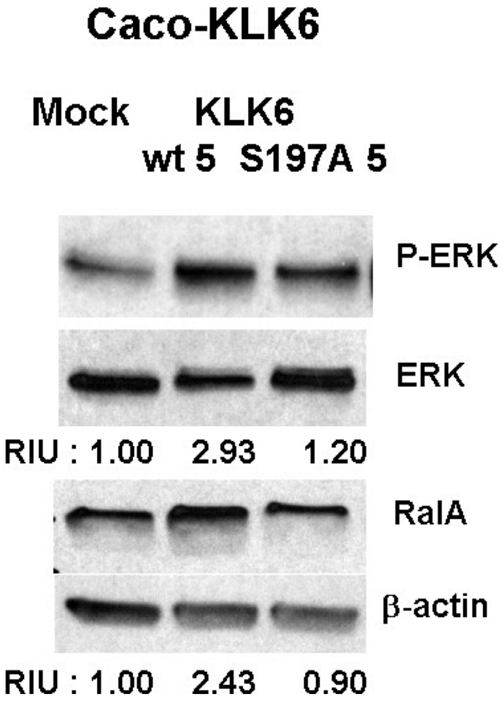

Supplementary Figure 3: Levels of phospho- ERK, total ERK and RalA proteins in Caco-KLK6 model. Samples were analyzed 48 hours after subculture. RIU: relative intensity units, quantification was done using Image J. Bands of the protein of interest were normalized to β-actin. The proteins sizes are indicated in kilodalton (kDa). Figure is representative of three independent experiments.

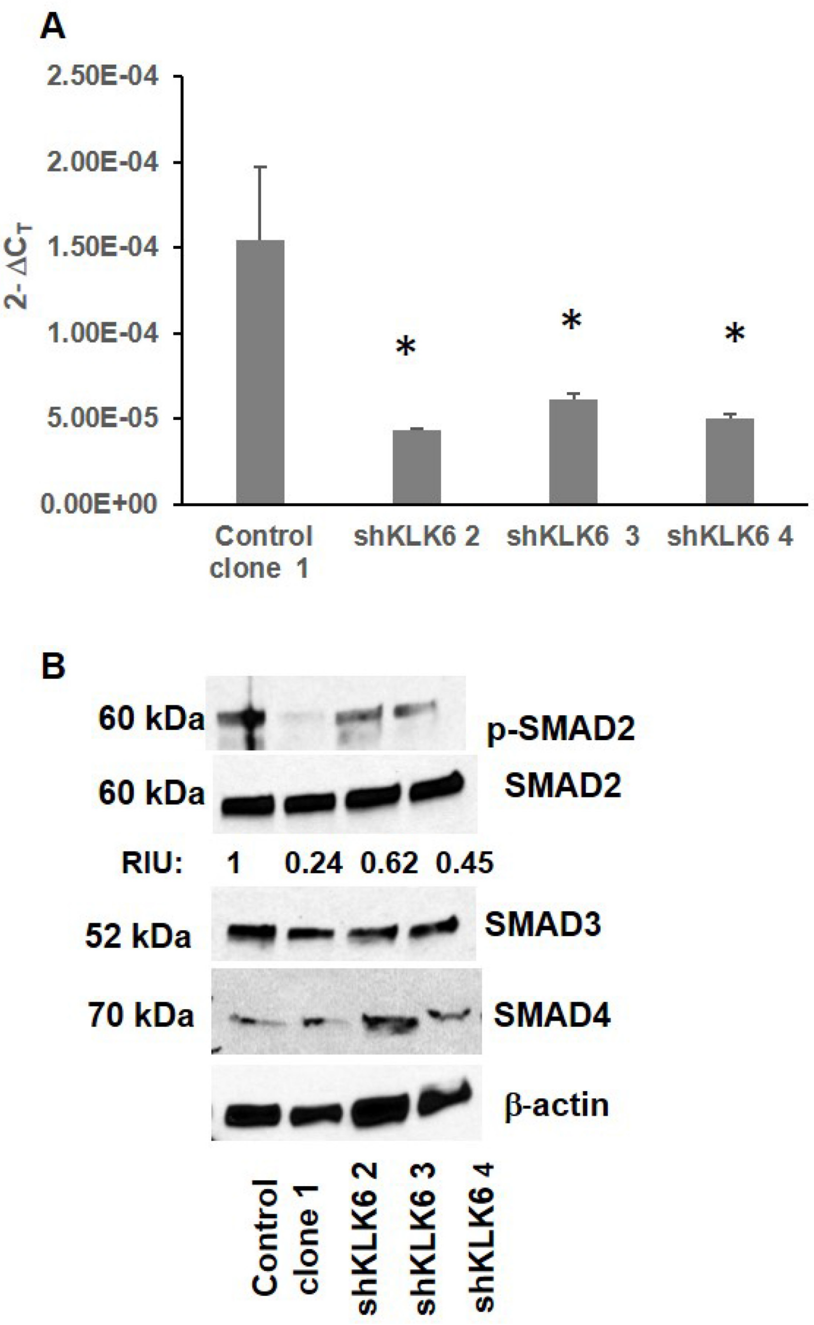
A
2.50E-04
2.00E-04
1.50E-04
1.00E-04
5.00E-05
0.00E+00
2- ΔCT
*
*
*
Control clone 1
shKLK6 2
shKLK6 3
shKLK6 4
B
60 kDa
p-SMAD2
60 kDa
SMAD2
RIU: 1 0.24 0.62 0.45
52 kDa
SMAD3
70 kDa
SMAD4
β-actin
Control clone 1
shKLK6 2
shKLK6 3
shKLK6 4

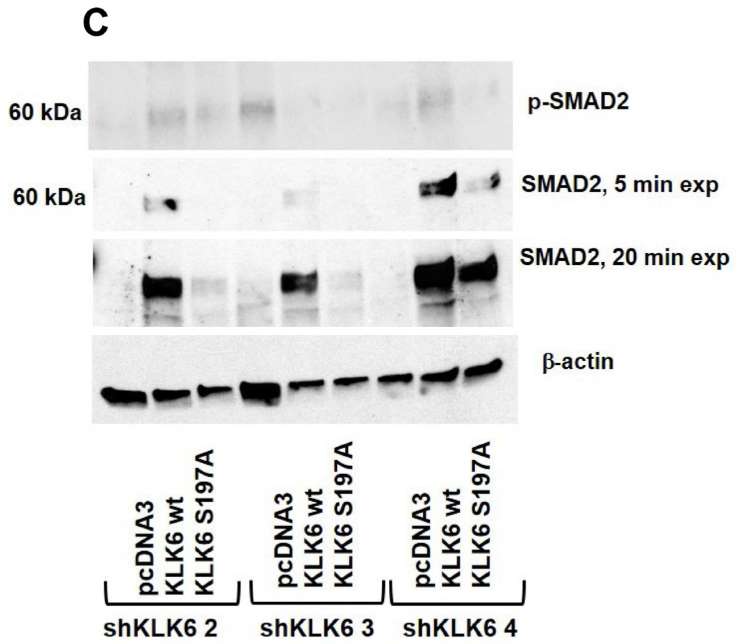

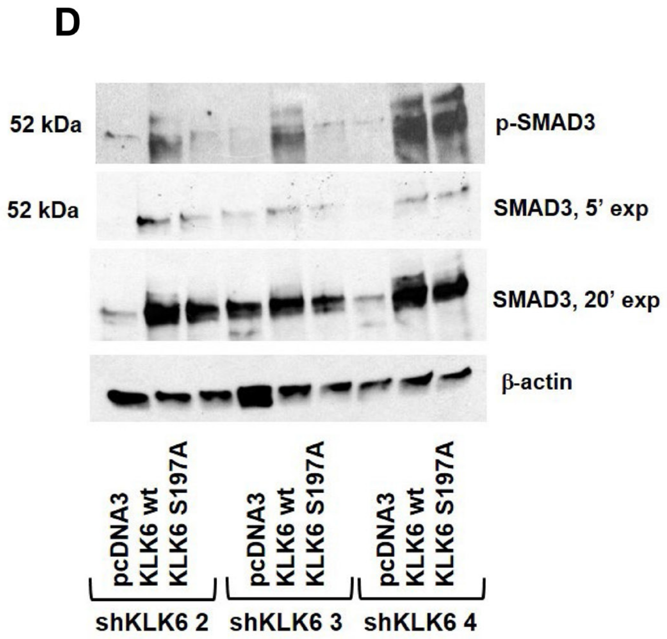

Supplementary Figure 4: Status of SMAD signaling in HCT116-shKLK6 cell model. (**A**) Analysis of TGF-β2 expression in HCT116 Control clone 1 and shKLK6 clones isogenic clones by qPCR; $^{*}p \leq 0.02$ (HCT116 Control clone 1 vs shKLK6 clones 2–4), (**B**) SMAD proteins expression in HCT116-shKLK6 cell model. RIU: relative intensity units, quantification was done using Image J. Bands of phosphorylated SMAD2 protein were normalized to the level of total SMAD2 protein. The protein sizes are indicated in kDa. Figure is representative of two independent experiments. (**C**) Expression of phosphorylated and total SMAD proteins in shKLK6 isogenic clones transiently transfected with KLK6 wt and KLK6S197A plasmids. The shKLK6 clones 2,3,and 4 were transiently transfected with pcDNA 3.1 vector or KLK6 wt and KLK6 S197A plasmids as described in “Materials and Methods” section, and were harvested 48 h after transfection for analysis of the levels of the total and phosphorylated SMAD2 (A) and the total and phosphorylated SMAD3 (**D**) proteins. β-actin was used as a loading control. The protein sizes are indicated in kDa. Figure is representative of two independent experiments.

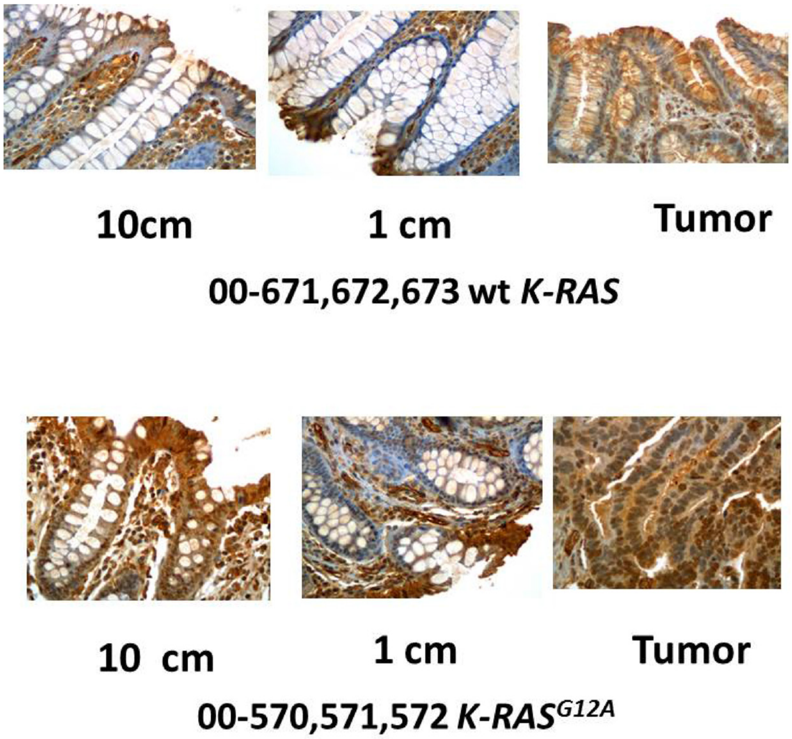

Supplementary Figure 5: Representative images of KLK6 IHC staining in the distant (10 cm away of a tumor), adjacent (1 cm away from tumor) and cancerous (tumor) tissues of 2 colon cancer patients (samples ID 00-671, 672, 673 with wild-type *K-RAS* status and 00-570, 571, 572 with *K-RASG*^{12A} status are shown).
